# Genome-Wide Identification and Expression Pattern of MYB Family Transcription Factors in *Erianthus fulvus*

**DOI:** 10.3390/genes14122128

**Published:** 2023-11-25

**Authors:** Xibing Rao, Zhenfeng Qian, Linyan Xie, Huaying Wu, Quan Luo, Qiyue Zhang, Lilian He, Fusheng Li

**Affiliations:** 1 College of Agronomy and Biotechnology, Yunnan Agricultural University, Kunming 650201, China; rxibing@163.com (X.R.); m18787436712@163.com (Z.Q.); xly1977151909@163.com (L.X.); why15912216153@163.com (H.W.); 18313390166@163.com (Q.L.); 18087167538@163.com (Q.Z.); 2 The Key Laboratory for Crop Production and Smart Agriculture of Yunnan Province, Kunming 650201, China; 3 Sugarcane Research Institute, Yunnan Agricultural University, Kunming 650201, China

**Keywords:** sugarcane, *Erianthus fulvus*, MYB transcription factor, gene expression

## Abstract

MYB family genes have many functions and are widely involved in plant abiotic-stress responses. *Erianthus fulvus* is an important donor material for stress-resistance genes in sugarcane breeding. However, the *MYB* family genes in *E. fulvus* have not been systematically investigated. In this study, 133 *EfMYB* genes, including 48 *Ef1R-MYB*, *84 EfR2R3-MYB* and 1 *Ef3R-MYB* genes, were identified in the *E. fulvus* genome. Among them, the *EfR2R3-MYB* genes were classified into 20 subgroups. In addition, these *EfMYB* genes were unevenly distributed across 10 chromosomes. A total of 4 pairs of tandemly duplicated *EfMYB* genes and 21 pairs of segmentally duplicated *EfMYB* genes were identified in the *E. fulvus* genome. Protein-interaction analysis predicted that 24 EfMYB proteins had potential interactions with 14 other family proteins. The *EfMYB* promoter mainly contains cis-acting elements related to the hormone response, stress response, and light response. Expression analysis showed that *EfMYB39*, *EfMYB84*, and *EfMYB124* could be significantly induced using low-temperature stress. *EfMYB30*, *EfMYB70*, *EfMYB81*, and *EfMYB101* responded positively to drought stress. ABA treatment significantly induced *EfMYB1*, *EfMYB30*, *EfMYB39*, *EfMYB84*, and *EfMYB130*. All nine genes were induced using MeJA treatment. These results provide comprehensive information on *EfMYB* genes and can serve as a reference for further studies of gene function.

## 1. Introduction

Due to their “fixed” growth characteristics, plants are usually susceptible to unfavorable environmental factors during growth. After a long period of domestication, agricultural plants have developed a unique regulatory mechanism to adapt to these stresses. Upon sensing stress signals, transcription factors further regulate the expression of functional genes that enable plants to generate specific metabolic and physiological responses to mitigate the effects of stress [1,2]. *MYB* is a very important class of genes in plants. The presence of the MYB structural domain at its N-terminus is a hallmark feature of the MYB family. Its C-terminus is highly differentiated, which also creates the diversity of MYB proteins [3,4]. The structural domains of *MYB* genes usually contain varying numbers of R-repeat sequences, and based on their number, the *MYB* family genes are categorized as *1R-MYB*, *R2R3-MYB*, *3R-MYB*, and *4R-MYB*. Each R-repeat sequence contains approximately 50–55 amino acids, forms three α-helices, has an HTH structure between the last two α-helices, and is involved in the NDA-binding process [5,6]. The *3R-MYB* gene is reportedly generated with the occurrence of R1 duplication within the *R2R3-MYB* gene. However, it has also been reported that the *R2R3-MYB* gene is generated due to loss of R1 within the *3R-MYB* gene [7].

*MYB* family genes are functionally diverse and play important roles in plant development [8,9,10,11]. For example, *AtMYB96* enhances plant drought tolerance by controlling epidermal wax biosynthesis through the ABA signaling pathway [12]. Overexpression of the *OsMYB48-1* gene in rice significantly enhances the tolerance of rice to mannitol and PEG [13]. The *FtMYB9* gene further reduces salt-stress damage by regulating the synthesis of proline [14]. *MdSIMYB1* enhances plant tolerance to salt, drought, and low temperature by upregulating the expression of stress-response genes (*NtDREB1A*, *NtERD10B*, and *NtERD10C*) in transgenic tobacco [15]. *ZmMYB-IF35* expression alleviates oxidative damage in *Zea mays* under low-temperature stress [16]. *OsMYB30* enhances the protective effect of cell membranes by regulating amylolysis and maltose accumulation, resulting in enhanced cold tolerance in rice. In addition, *OsMYB30* plays an important role in the resistance of rice brown planthopper mediated by the phenol propane pathway [17,18]. *MdMYB30* binds to the promoter of the wax synthesis-related gene *MdKCS1* and activates its transcription to enhance the biosynthesis of epidermal waxes to regulate plant resistance to pathogens [19].

*Sugarcane* (*Saccharum officinarum* L.) is one of the most important cash crops and provides 80% of the world’s sugar and 90% of China’s sugar. Additionally, sugarcane is a promising renewable-energy crop with significant development potential [20]. However, in the region of Yunnan Province, China, adversities such as drought and low temperature often hinder the growth and development of sugarcane, and the precise and effective enhancement of sugarcane resistance has become an important direction for sugarcane breeding at this stage. *E. fulvus*, which is a closely related wild species of sugarcane, is characterized by high brix and easy flowering and exhibits a high degree of stress tolerance, which are traits that are valuable for sugarcane breeding [21]. Therefore, the excellent resistance genes in *E. fulvus* should be fully explored and utilized for improving the resistance of sugarcane in a targeted manner.

Although *E. fulvus* is very important, few studies have investigated *MYB* family genes in *E. fulvus*. The number of members, gene structure, evolutionary features, gene expression properties, and functions of the *MYB* family of genes in *E. fulvus* are unknown. Thus, we identified *MYB* family genes in the whole genome of *E. fulvus* and systematically analyzed the gene structure, conserved structural domains, chromosomal localization, promoter cis-acting elements, gene duplication, and protein-interaction relationships. In addition, we investigated the effects of low temperature, drought stress, and ABA and MeJA treatments on *EfMYB* gene expression. These results can enable us to obtain a better systematic understanding of the *EfMYB* family of genes and lay the foundation for our subsequent studies on *EfMYB* gene function, regulatory mechanisms, and transgenic breeding of sugarcane.

## 2. Materials and Methods

### 2.1. Identification of EfMYB Genes

The *E. fulvus* genome information and related files were downloaded from the *E. fulvus* Genome Database (http://efgenome.ynau.edu.cn/, accessed on 12 April 2023) (the Genome Database includes datasets such as *E. fulvus* genome-sequence data, genome annotation files, coding protein files, resequencing data, and low-temperature transcriptome data for *E. fulvus*) [22]. The Hidden Markov Model (HMM) of the MYB structural domain (PF00249) was obtained using the Protein Families Database (http://pfam.xfam.org/, accessed on 12 April 2023) [23]. Gene family identification and analysis were performed using Docker (version 4.18.0) tools. The list of genes containing MYB structures in the *E. fulvus* genome was obtained using Hmmsearch, and the structural domain information for the first 100 Hmmsearch outputs was intercepted to construct an *E. fulvus*-specific Hmm model with an EValue < 0.001 and perform a second search. In addition, a search of EfMYB proteins was conducted using the BLAST program with sorghum MYB proteins (Obtained from NCBI (https://www.ncbi.nlm.nih.gov/), accessed on 12 April 2023) as query sequences (EValue < 0.001). The obtained protein sequences were uploaded to NCBI-CDD (https://www.ncbi.nlm.nih.gov/cdd, accessed on 14 April 2023), SMART (http://smart.embl-heidelberg.de/, accessed on 14 April 2023), and Protein Family (http://pfam.xfam.org/, accessed on 14 April 2023) databases for structural-domain confirmation, and the correct EfMYB family proteins were ultimately obtained [24,25,26].

### 2.2. Phylogenetic Tree Analysis

Multiple sequence comparison of the EfR2R3-MYB proteins and 126 *Arabidopsis thaliana* AtR2R3-MYB proteins was carried out using MEGA 7.0 software with the neighbor-joining method (NJ method) and a bootstrap value of 1000 to construct the phylogenetic tree [27,28].

### 2.3. Protein-Properties, Conserved-Motifs, and Gene-Structure Analysis

The protein properties and subcellular localization of *EfMYB* family members were predicted using the online tools ExPASy (https://web.ExPASy.org/protparam/, accessed on 17 April 2023) and WoLF PSORT (https://wolfpsort.hgc.jp/, accessed on 17 April 2023), respectively [29]. The online tool MEME (Version 5.5.4) was then used to generate 20 conserved motifs of the EfMYB protein based on a minimum sequence length of 6 and a maximum length of 100 (https://meme-suite.org/meme/, accessed on 17 April 2023) [30]. The R2 and R3 repeat motifs of the EfR2R3-MYB protein were generated using WebLogo (https://weblogo.berkeley.edu/logo.cgi, accessed on 17 April 2023) to further characterize the conserved structural domains of MYB [31]. Finally, the conserved motifs and gene structure of the *EfMYB* genes were visualized using TBtools [32].

### 2.4. Chromosomal-Location, Gene-Duplication, and Synteny Analysis of EfMYB Genes

Chromosomal localization data of the *EfMYB* genes were extracted using Docker and visualized using MapChart [33]. The MCScan tool was used to analyze the tandem-duplication (TD) and segmental-duplication (SD) events of *EfMYB* genes and for the synteny analysis of MYB genes between *E. fulvus* and *A. thaliana*, *Zea mays*, *Oryza sativa*, *Saccharum spontaneum,* and *Sorghum bicolor* [34]. Finally, we analyzed nonsynonymous (ka) and synonymous (ks) substitutions in the *EfMYB* genes that underwent replication events using the KaKs_Calculator 2.0 tool [35].

### 2.5. Identification of Cis-Acting Elements in EfMYB Genes

The promoter sequence 2000 bp upstream of the EfMYB gene sequence was extracted from the genome file using the Docker tool. The cis-acting elements were then predicted using the PlantCARE online tool (http://bioinformatics.psb.ugent.be/webtools/plantcare/html/, accessed on 25 April 2023), and their potential related functions were speculated [36].

### 2.6. Protein-Interaction Network and RNA-Seq Data Analysis of EfMYB Genes

EfMYB proteins were uploaded to OrthoVenn2 to identify EfMYB proteins homologous to *A. thaliana* MYB proteins (https://orthovenn2.bioinfotoolkits.net/home, accessed on 28 April 2023), and the STRING tool was then used to generate the protein-interaction network of EfMYB (https://cn.string-db.org/, accessed on 28 April 2023) [37]. Finally, the Cytoscape v3.8.2 tool was used to further edit the image [38]. RNA-seq data of *E. fulvus* leaves under low-temperature-stress (4 °C) treatment (0 h, 24 h, 72 h) were downloaded from the database. The expression heatmap of *EfMYB* genes was constructed using the TBtools tool.

### 2.7. Material Processing and Gene Expression

First, the *E. fulvus* clones were planted in pots, and the following stress treatments were executed after the plants had grown five leaves. The low-temperature-stress treatments were conducted as follows. The plant material was transferred to a cold incubator at 4 °C and sampled after 0 h (CK), 24 h, and 72 h of stress treatment. Drought-stress treatments were conducted by stopping the supply of water to the plants, and samples were taken after 0 d (CK), 3 d, 6 d, and 9 d of the stress treatment. The soil water content at each drought-stress time point was 70 ± 5%, 50 ± 5%, 30 ± 5%, and 15 ± 5%. For exogenous hormone treatments, *E. fulvus* asexual tissue culture seedlings were sprayed with abscisic acid (ABA) and methyl jasmonate (MeJA) solutions at a concentration of 100 µM. Samples were collected after 0 h (CK), 6 h, and 12 h of treatment. After performing the above treatments, the samples collected were all of the first fully expanded leaves of the plant material, counted from top to bottom. RNA was extracted using TRIzol reagent from Tiangen Biochemical Technology (Beijing, China), and the quality and concentration of RNA were then detected using 1% agarose gel electrophoresis and UV spectrophotometry. Finally, the better-quality RNA was reverse transcribed into cDNA. qRT-PCR analysis was performed with an Applied Biosystems ABI 7500 instrument using 2 × RealStar Fast SYBR qPCR Mix (GenStar) (Table S8). We used the *25S rRNA* gene as an internal reference to calculate the expression levels of the *EfMYB* genes with the 2^−ΔΔct^ method [39].

## 3. Results

### 3.1. Identification and Protein Characterization of EfMYB Genes

A total of 133 *EfMYB* genes were identified in the *E. fulvus* genome, including 48 *Ef1R-MYB*, 84 *EfR2R3-MYB*, and 1 *Ef3R-MYB* genes (Table S1). The *EfMYBs* were named *EfMYB1*~*EfMYB133* based on their arrangement order on chromosomes. The predicted protein physicochemical properties are shown in Table S2. The molecular weight (MW) was 12,252 Da (*EfMYB45*)~231,536.9 Da (*EfMYB106*), the isoelectric point (pI) was 4.38 (*EfMYB82*)~11.49 (*EfMYB67*), the amino acid lengths ranged from 110 to 2143 aa, and the average amino acid lengths for MW and pI were 448.37 aa, 48,681.63 Da and 8.01, respectively. The predicted subcellular localization of the *EfMYBs* showed that a few members were located in chloroplasts (7.5%), while the majority were located in the nucleus (92.5%) (Table S2), indicating that these EfMYB transcription factors may play regulatory roles in the chloroplast and nucleus, respectively.

### 3.2. Phylogenetic, Conserved-motif, and Gene-Structure Analyses of the EfMYBs

**Figure 1:**
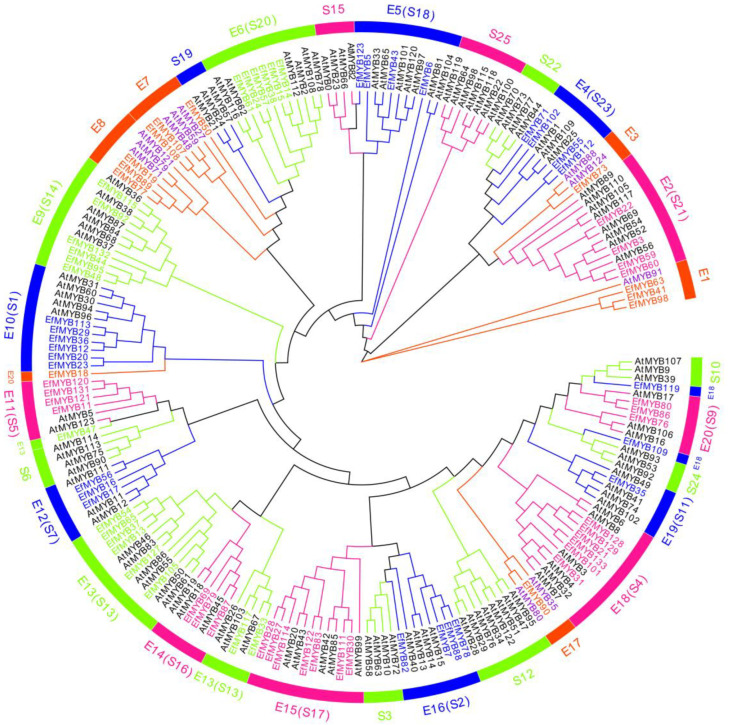
Phylogenetic tree of R2R3-MYB proteins between *E. fulvus* and *A. thaliana*. *EfR2R3-MYB* is marked in color. *AtR2R3-MYB* is marked in black. The violet marking for the *AtR2R3-MYB* genes indicates that they belong to the unclassified-gene category.

As previously reported [40], compared with other types of *EfMYB* genes, *EfR2R3-MYB* genes accounted for the largest percentage (63.16%) of the *EfMYB* family. To analyze the characteristics of *EfR2R3-MYB* proteins, we built a phylogenetic tree of 84 EfR2R3-MYB proteins with 126 AtR2R3-MYB proteins (Figure 1). *EfR2R3-MYB* was divided into 20 subgroups (E1~E20) based on multiple sequence alignment. Among them, E1, E2 (S21), E3, E4 (S23), E5 (S18), E6 (S20), E7, E8, E9 (S14), E10 (S1), E11 (S5), E12 (S7), E13 (S13), E14 (S16), E15 (S17), E16 (S2), E17, E18 (S4), E19 (S11), and E20 (S9) all contained *E. fulvus* and *A. thaliana R2R3-MYB* genes, whereas S22, S25, S15, S19, S6, S3, S12, S24, and S10 contained only the *AtR2R3-MYB* genes. Overall, subgroups E13 (S13) and E15 (S17) had the highest number of *EfR2R3-MYB* genes (nine and seven, respectively); subgroups E3 and E17 had the fewest number of *EfR2R3-MYB* genes (only one in both groups); and most of the other subgroups contained three to four genes.

**Figure 2:**
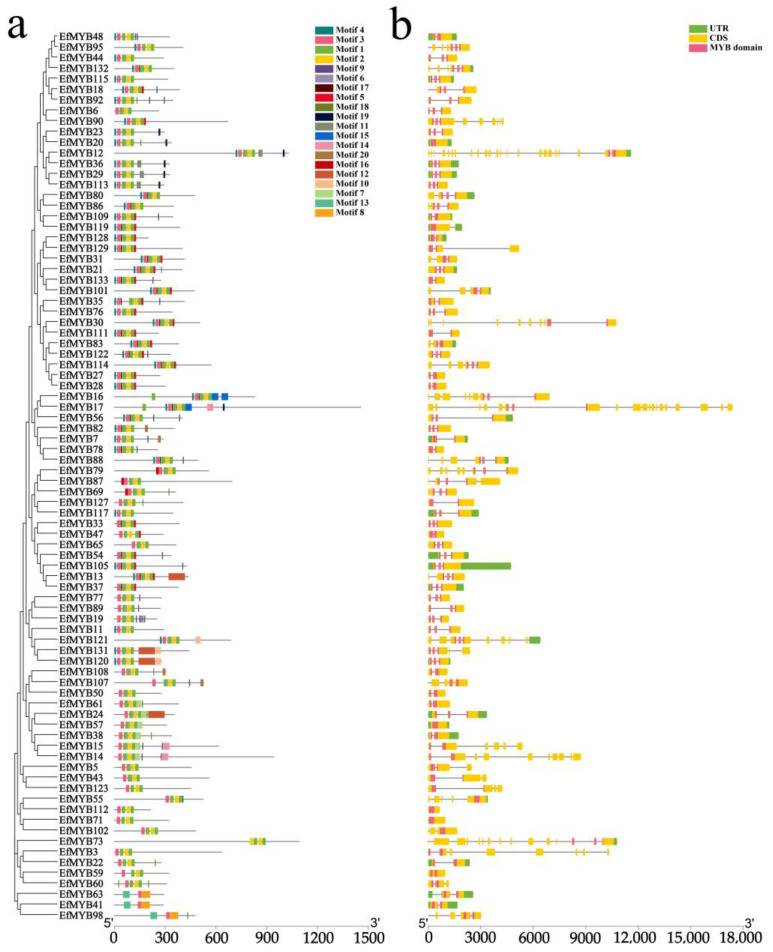
Conserved motifs and gene structure of *EfR2R3-MYB* genes. (**a**) The motif compositions of the *EfR2R3-MYB*. Colored boxes represent different motifs. (**b**) Structural characterization of the *EfR2R3-MYB*. The green, yellow, and pink boxes represent the UTR, CDS, and MYB domains, respectively.

**Figure 3:**
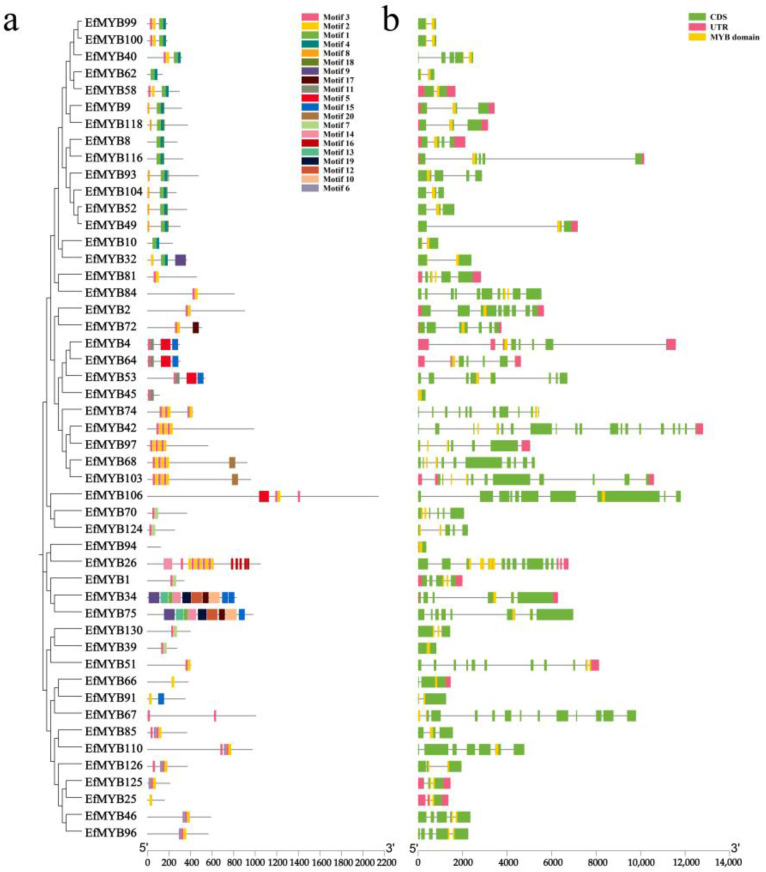
Conserved motifs and gene structure of *Ef1R-MYB* and *Ef3R-MYB* genes. (**a**) The motif compositions of the *Ef1R-MYB* and *Ef3R-MYB*. Colored boxes represent different motifs. (**b**) Structural characterization of the *Ef1R-MYB* and *Ef3R-MYB*. The green, yellow, and pink boxes represent the CDS, MYB domains, and UTR, respectively.

It is generally believed that motifs may contain specific binding sites and be involved in specific biological functions. We predicted 20 motifs (Motif 1~Motif 20) for *EfMYB* family genes (Table S4). The results showed that almost all the *EfR2R3-MYB* genes contained Motif 3, Motif 2, and Motif 1, and more than half of the *EfR2R3-MYB* genes contained Motif 4. In addition, only Motif 15 appeared in *EfMYB16* and *EfMYB17*, and only Motif 14 appeared in *EfMYB14* and *EfMYB15*. Motif 16 and Motif 13 were unique to the E14 (S16) subgroup and E1 subgroup, respectively (Figure 2a). In summary, most *EfR2R3-MYB* genes in the same subgroup usually have similar motif types, and motifs between different subgroups may lead to differences in *EfMYB* gene function. Compared to *EfR2R3-MYB*, *Ef1R-MYB* contained a markedly smaller number of motifs (Figure 3a). However, EfMYB94 did not generate motif under the parameters we set, which may be due to sequence differences between EfMYB94 and other proteins. Interestingly, there were multiple repetitions of Motif 3, Motif 2, and Motif 16 in *Ef3R-MYB* (*EfMYB26*). Motifs can bind to transcription factors, regulate gene expression, or participate in protein-protein interactions. This suggests that the repeated occurrence of these motifs in *EfMYB26* may be recognized by specific transcription factors for binding to regulatory regions of genes and thereby exert certain unique biological effects [41]. We further analysed the core motifs of the EfR2R3-MYB structural domain and found that many amino acid residues were conserved in the R2 and R3 repeat sequences. The R2 and R3 repeat motifs contained 3 and 2 conserved tryptophan residues (W), respectively (Supplementary Figure S1).

Diversity in gene structure may be a basis for the evolution of gene families. To understand the structural characteristics of *EfMYB* family genes, we analyzed the exon and intron distributions of *EfR2R3-MYB*, *Ef1R-MYB,* and *Ef3R-MYB*. The results showed that the number of exons in the *EfR2R3-MYB* genes ranged 1~23, with 36.9% of the members containing two introns and 23.8% containing one intron. Notably, *EfMYB20*, *EfMYB57*, *EfMYB112*, *EfMYB71*, and *EfMYB59* contained no introns, while *EfMYB12* contained 22 introns (Figure 2b). The numbers of introns in the *Ef1R-MYB* genes were mainly one, two or four. However, *EfMYB42* had the highest number of introns (19), and three genes (*EfMYB45*, *EfMYB94*, *EfMYB39*) had no introns (Figure 3b). Many *EfMYB* genes demonstrated a domain with a cross-intron structure (Figure 2b and Figure 3b). These results indicated that the structures of the *EfMYB* genes were diverse. 

### 3.3. Chromosomal-Distribution and Gene-Duplication Analysis of EfMYB Genes

**Figure 4:**
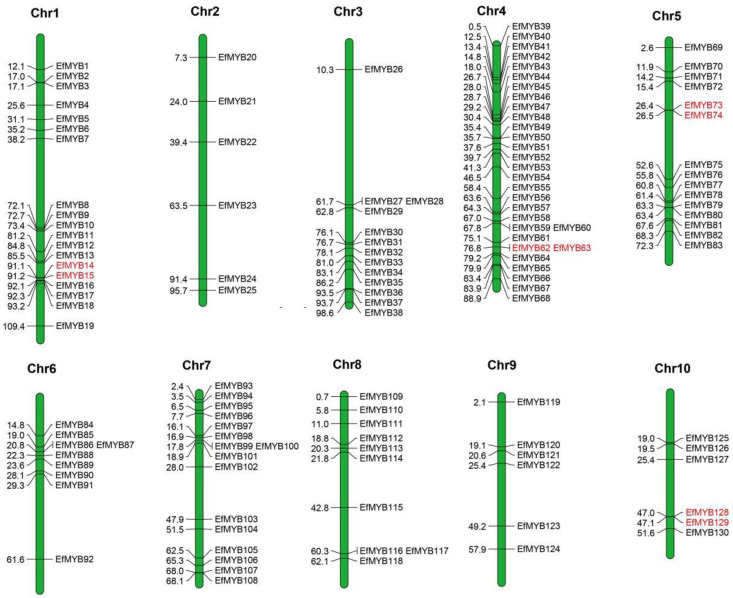
Chromosomal-localization and tandem-duplication analysis of the *EfMYB* gene. The red genes on the chromosome scaffold represent *EfMYB* tandem-duplicated gene pairs.

The results showed that three genes (*EfMYB131*, *EfMYB132*, and *EfMYB133*) were not localized on any chromosome, and the remaining 130 *EfMYB* genes were unevenly distributed on Chr1-Chr10. The highest number of genes was distributed on Chr4 (30), followed by Chr1 and Chr7 (19 and 16). The lowest number of genes (6) was found on Chr2, Chr9, and Chr10 (Figure 4).

**Figure 5:**
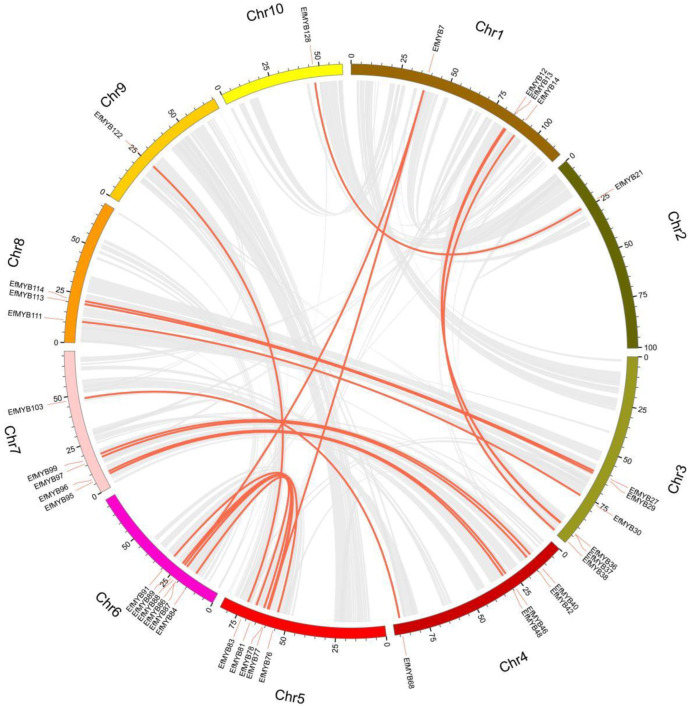
Segmental-duplication analysis of the *EfMYB* gene within the genome. The gray line represents the collinear region in the *E. fulvus* genome, and the orange line represents duplicated *EfMYB* gene pairs.

Tandem duplications and segmental duplications are events that occur frequently in plant genomes, and they contribute to the expansion of gene families, which gives rise to new members and functions that drive plant evolution [42]. To investigate the expansion of *EfMYB* family genes, we analyzed tandem duplications and segmental duplications of *EfMYB* genes using the MCScanX tool (Table S3). As shown in Figure 4, four tandem duplicate gene pairs were found on Chr1 (*EfMYB14*-*EfMYB15*), Chr4 (*EfMYB62*-*EfMYB63*), Chr5 (*EfMYB73*-*EfMYB74*), and Chr10 (*EfMYB128*-*EfMYB129*). In addition, we identified 21 *EfMYB* segmental-duplication gene pairs (Figure 5). The *EfMYB* gene segmental-duplication events occurred mainly in Chr3 (6 genes), Chr4 (5 genes), Chr5 (5 genes), Chr6 (6 genes), and Chr7 (5 genes), indicating that segmental duplication and tandem duplication events are important factors driving the expansion of *EfMYB* genes in *E. fulvus*.

We further calculated the Ka/Ks values of these duplicated gene pairs to more clearly analyze the evolutionary process of the *EfMYB* genes (Table S3). The results showed that the Ka/Ks values ranged from 0.07 to 1027. A total of 58.33% of the gene pairs had values < 1, indicating that these genes may have evolved under purifying selection pressure, whereas another 41.67% of the genes may have evolved under positive selection pressure after replication (Ka/Ks > 1). In addition, we found that the timing of gene duplication events was 17.83 Mya~204.86 Mya.

### 3.4. Synteny Analysis

**Figure 6:**
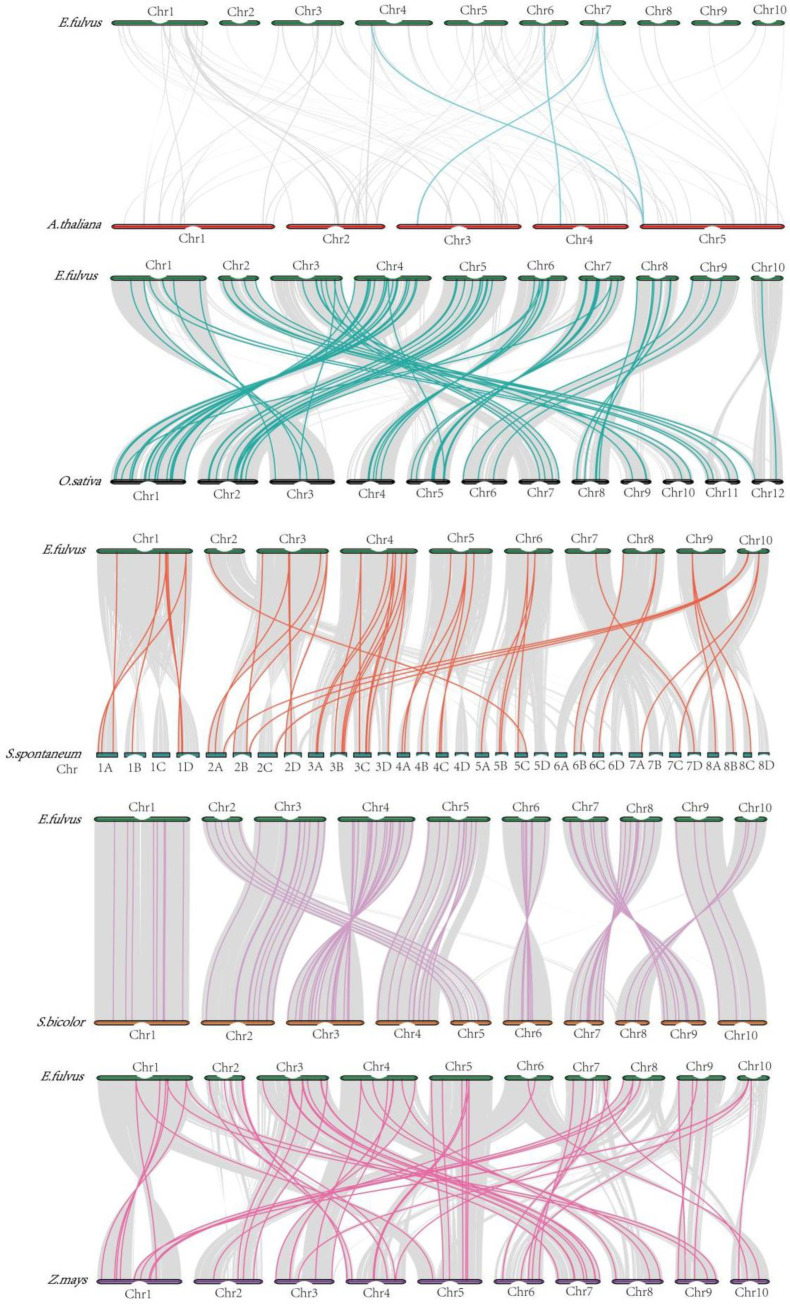
Synteny analysis of *EfMYB* genes with *MYB* genes from other species. The grey background represents collinear regions within the genomes of sorghum and the exemplified species, whereas the colored lines represent *MYB* gene pairs with collinearity.

We performed *MYB* gene synteny analysis of five representative plants (*S. bicolor*, Z. mays, *A. thaliana*, *O. sativa*, and *S. spontaneum*) with *E. fulvus* to better understand the evolutionary features of *MYB* genes (Table S5; Figure 6). The highest number of *MYB* homologous genes with sucrose was found in *S. bicolor* (84 pairs), followed by *O. sativa* (74 pairs). However, the lowest numbers of *MYB* homologues were found in *E. fulvus* and *A. thaliana* (3 pairs). Notably, 13 *EfMYB* genes (*EfMYB13*, *EfMYB19*, *EfMYB20*, *EfMYB36*, *EfMYB38*, *EfMYB28*, *EfMYB43*, *EfMYB61*, *EfMYB56*, *EfMYB77*, *EfMYB88*, *EfMYB91*, and *EfMYB109*) have syntenic gene pairs with rice, sorghum, *Saccharum spontaneum* and maize *MYB* genes. This finding shows that *MYB* family genes are highly conserved in Gramineae.

### 3.5. Cis-Acting Regulatory Elements in the Promoters of EfMYBs

**Figure 7:**
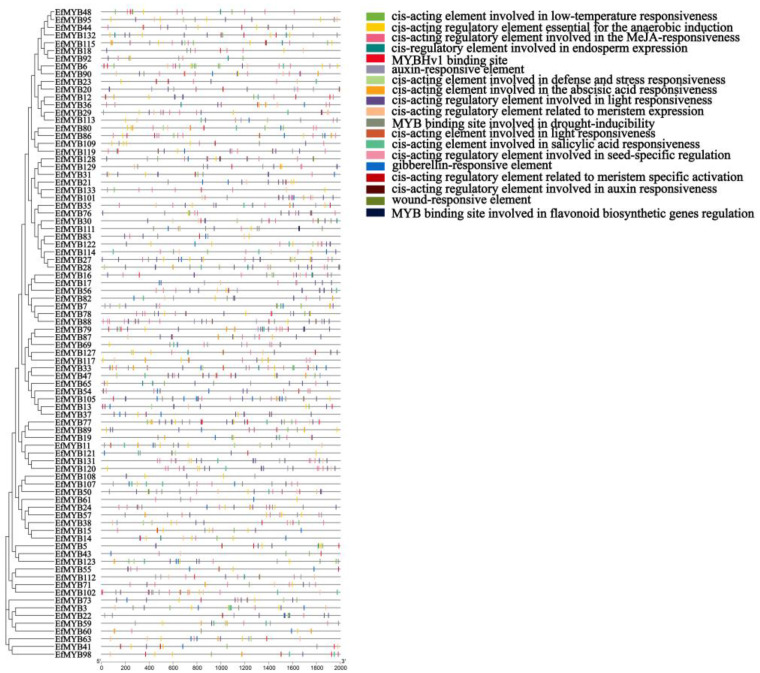
Cis-regulatory-element prediction of *EfR2R3-MYB* genes. Each cis-regulatory element is distinguished using a box of a different color.

**Figure 8:**
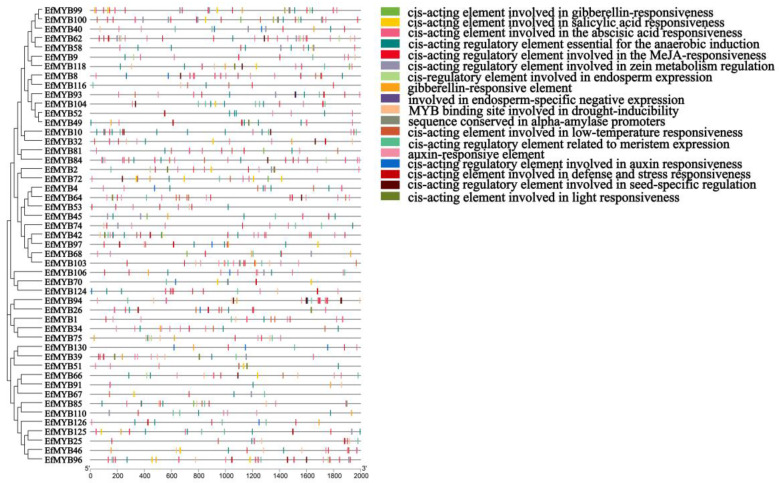
Cis-regulatory-element prediction of *Ef1R-MYB* and *Ef3R-MYB* genes. Each cis-regulatory element is distinguished using a box of a different color.

The study of promoter cis-acting elements is crucial for understanding the transcriptional regulatory properties of *EfMYB* genes and inferring their potential functions. Therefore, we predicted numerous elements using PlantCARE that may regulate *EfMYB* genes (Table S6). The cis-acting elements in the *EfMYB* promoter can be mainly classified into several categories related to plant secondary metabolism, environmental-stress response, light response, hormonal response and plant development. A total of 48 low-temperature-related cis-acting elements (LTR) were found in the promoter of *EfR2R3-MYB*, suggesting that they may play a role in *E. fulvus* under cold stress. Some MYB-binding sites related to the response to drought and the regulation of flavonoid biosynthesis were found in the promoters of *EfMYB46*, *EfMYB105*, *EfMYB87,* and *EfMYB25*. In addition, some regulatory elements involved in seed-specific regulation and meristem expression were also discovered. A total of 64 auxin-related elements, 58 gibberellin-response-related elements, and 41 salicylic-acid-related elements were identified. Notably, we found that regulatory elements associated with MeJA and ABA were most common in *EfR2R3-MYB*, with 374 and 292, respectively (Figure 7). Similar phenomena were also found in the *Ef1R-MYB* genes (Figure 8). The *EfMYB* genes are perhaps involved in the regulation of various types of plant responses through the hormone signaling pathway.

### 3.6. Protein-Interaction-Network Analysis of the EfMYB Gene

**Figure 9:**
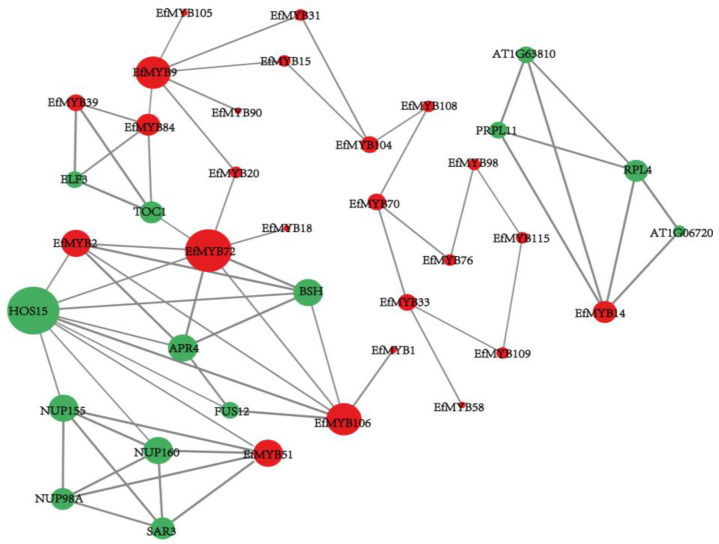
Analysis of the EfMYB protein-interaction network. Red color represents EfMYB family proteins, and other family proteins are indicated in green. The thickness of the connecting line represents the strength of the interaction between proteins.

To explore the interaction relationship of EfMYB proteins, *Arabidopsis* proteins homologous to EfMYB proteins were mapped in OrthoVenn2, and protein-interaction networks were further constructed (Table S7). The protein-interaction network consists of a total of 24 EfMYB family proteins and 14 other family proteins. Among them, EfMYB9, EfMYB72, and EfMYB106 interacted with many EfMYB family proteins, and EfMYB14, EfMYB51, and EfMYB2 interacted with AT1G06720, AT1G63810, RPL4, PRPL11, SAR3, NUP98A, NUP155, NUP160, HOS15, BSH, and ARP4 (Figure 9).

### 3.7. Effects of Low-Temperature and Drought Stress on EfMYB Gene Expression

**Figure 10:**
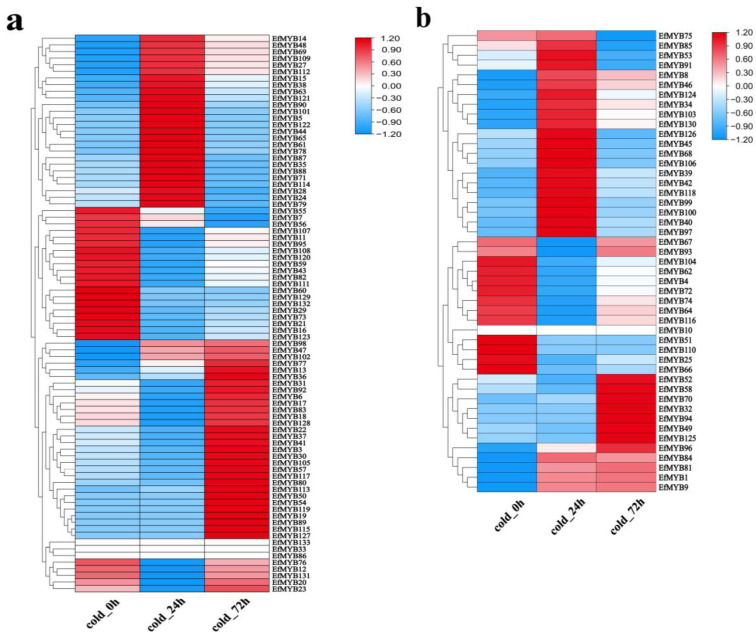
Heatmap of *EfMYB* gene expression in leaves under low-temperature stress. (**a**) Heatmap of *EfR2R3-MYB* gene expression. (**b**) Heatmap of *Ef1R-MYB* gene expression. A heatmap was generated using FPKM values. The blue, white, and red boxes represent low, moderate, and high gene expression, respectively.

We used TBtools to map the heatmap of *EfMYB* gene expression in *E. fulvus* leaves under low-temperature stress (0 h, 24 h, and 72 h) (Figure 10). A total of 20 *EfR2R3-MYB* genes and 11 *Ef1R-MYB* genes exhibited downregulation of expression after low-temperature stress. After 24 h of low-temperature stress, 26 *EfR2R3-MYB* genes and 20 *Ef1R-MYB* genes showed upregulated expression. After 72 h of low-temperature stress, 32 *EfR2R3-MYB* genes and 12 *Ef1R-MYB* genes showed upregulated expression. Among them, nine *EfMYB* genes (*EfMYB1*, *EfMYB30*, *EfMYB39*, *EfMYB70*, *EfMYB81*, *EfMYB84*, *EfMYB101*, *EfMYB124*, and *EfMYB130*) were strongly induced by low-temperature stress. The results imply that all of these genes, which showed significantly upregulated or downregulated expression under cold stress, may play important roles in the low-temperature response of *E. fulvus*.

**Figure 11:**
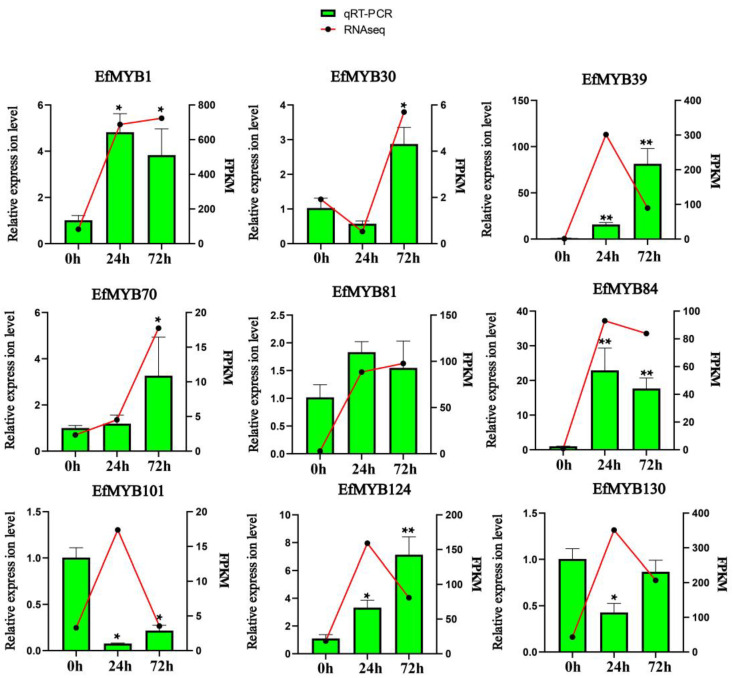
Expression profile of *EfMYB* genes under low-temperature stress. All graphs were generated using the means of three independent replicate experiments, and significant (*p* < 0.05) and highly significant (*p* < 0.01) increases or decreases relative to the CK group are indicated using * and **, respectively. The line graph represents the FPKM values.

We used qRT-PCR to further verify whether these nine *EfMYB* genes are cold-responsive genes (Figure 11). The results showed that the expression of three genes (*EfMYB30*, *EfMYB70*, *EfMYB84*) could be significantly induced by low temperature, and the expression trend was consistent with the FPKM values. In addition, four other genes *(EfMYB1*, *EfMYB39*, *EfMYB81*, *EfMYB124*) whose expression could also be induced by low temperature were also identified, but their expression trend was not completely consistent with the FPKM values. It is hypothesized that these seven *EfMYB* genes play important roles in early or late events of the cold stress response in *E. fulvus*. In contrast, the expression of *EfMYB101* and *EfMYB130* was not induced by low temperature.

**Figure 12:**
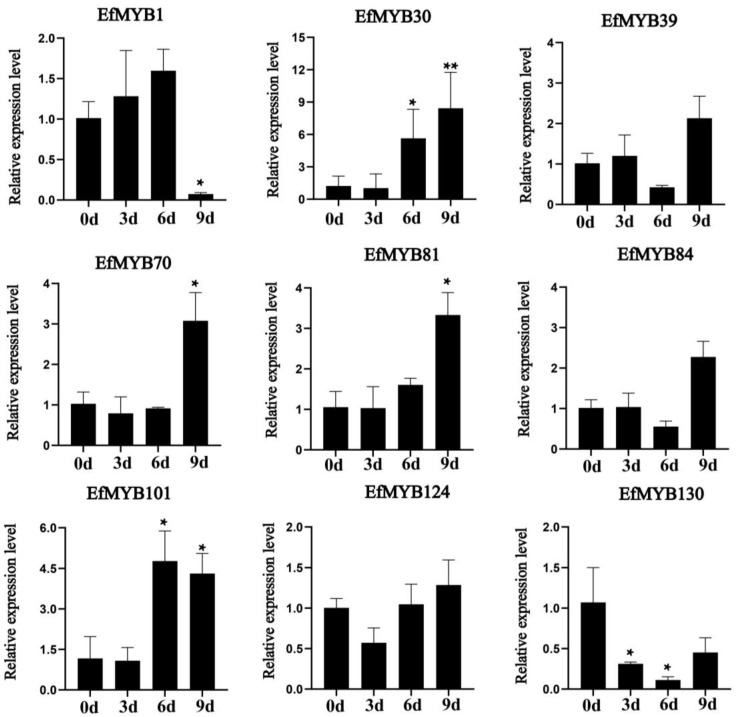
Expression profile of *EfMYB* genes under drought stress. All graphs were generated using the means of three independent replicate experiments, and significant (*p* < 0.05) and highly significant (*p* < 0.01) increases or decreases relative to the CK group are indicated with * and **, respectively.

We then analyzed the effects of drought stress on the expression of these nine *EfMYB* genes (Figure 12). Drought stress significantly induced the expression of *EfMYB30*, *EfMYB70*, *EfMYB81,* and *EfMYB101*. After 9 d of stress, the expression levels of *EfMYB39*, *EfMYB124,* and *EfMYB84* were also somewhat increased compared with those in the CK group. However, the expression levels of *EfMYB1* and *EfMYB130* were downregulated after drought stress.

### 3.8. Effects of ABA and MeJA Treatments on EfMYB Gene Expression

**Figure 13:**
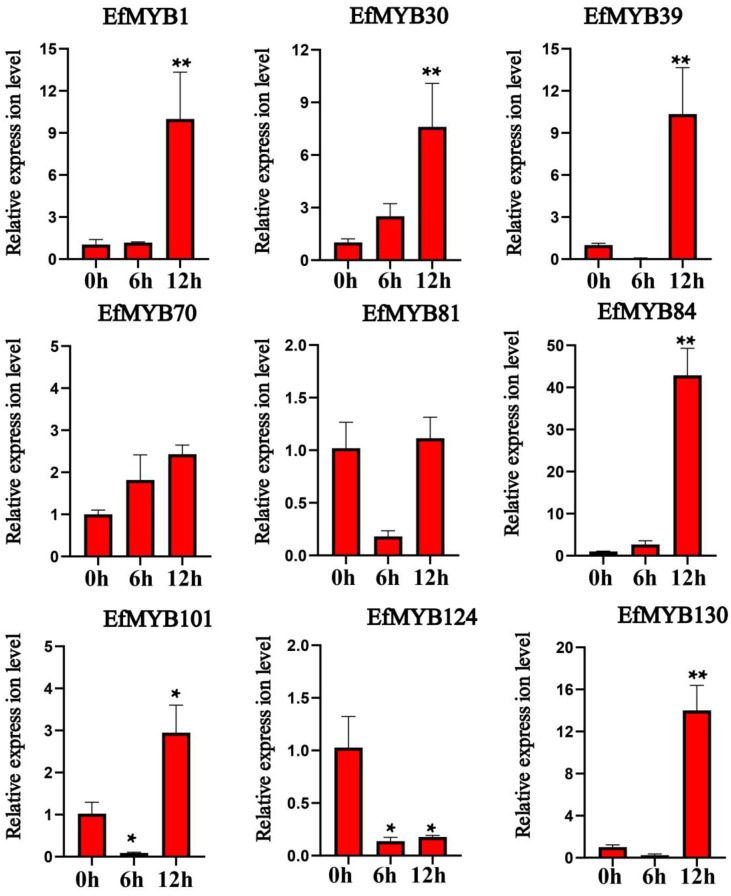
Expression profile of *EfMYB* genes under ABA treatment. All graphs were generated using the means of three independent replicate experiments, and significant (*p* < 0.05) and highly significant (*p* < 0.01) increases or decreases relative to the CK group are indicated with * and **, respectively.

**Figure 14:**
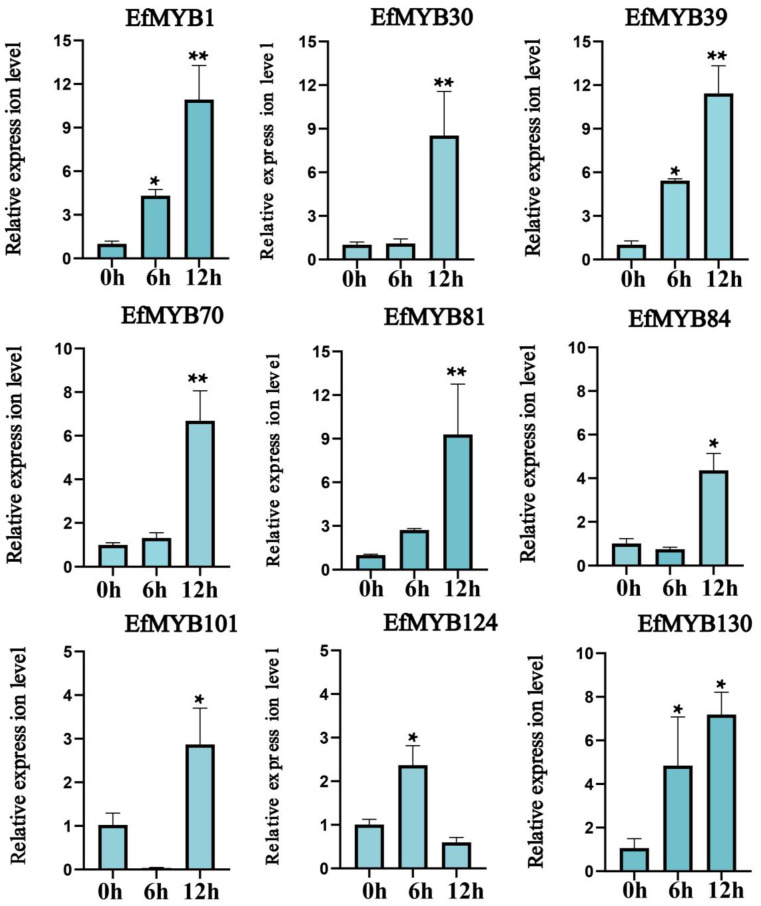
Expression profile of the *EfMYB* genes under MeJA treatment. All graphs were generated using the means of three independent replicate experiments, and significant (*p* < 0.05) and highly significant (*p* < 0.01) increases or decreases relative to the CK group are indicated with * and **, respectively.

Related studies have reported that *MYB* genes regulate environmental-stress responses in plants through hormone signaling pathways [13]. Our previous analysis showed that MeJA- and ABA-related regulatory elements are most common in the promoters of *EfMYB* genes. Therefore, we further investigated the effects of ABA and MeJA treatments on *EfMYB* gene expression. We found that five genes, *EfMYB1*, *EfMYB30*, *EfMYB39*, *EfMYB84*, *EfMYB101,* and *EfMYB130*, were significantly upregulated with ABA treatment for 12 h. ABA treatment had no significant effect on the expression of *EfMYB70* and *EfMYB81,* but significantly downregulated the expression of *EfMYB124* (Figure 13). The expression of all nine *EfMYB* genes was significantly induced with MeJA treatment. Except *EfMYB124*, which reached its maximal expression after 6 h of MeJA treatment, the expression of the remaining eight genes was highest after 12 h of treatment (Figure 14).

## 4. Discussion

The *MYB* family genes are functionally diverse, and the members of the family are widely distributed across various plant species. Since the discovery of the first plant *MYB* gene (c1) [43], with the continuous development of sequencing technology, *MYB* genes have been identified in an increasing number of species, including *A. thaliana* (198), rice (155), tomato (127), and potato (158) [44,45,46,47]. Researchers recently completed whole-genome sequencing of *E. fulvus*, which provides favorable support for us to carry out *EfMYB* gene identification, structure, and evolutionary characterization [48]. Based on this recent work, we identified a total of 133 *EfMYB* genes from the whole genome of *E. fulvus* (genome size: 902 Mb). The distribution of *MYB* genes in plants is not directly related to the size of the plant’s own genome. For example, *A. thaliana* (genome size: 125 Mb) has a relatively large number of *MYB* genes (198), but sorghum (genome size: 818 Mb) has only 145 *MYB* genes [49]. Consistent with other reports [50], in plants, the number of *EfR2R3-MYB* subgroup genes was also the highest among all *EfMYB* genes. However, we did not find *Ef4R-MYB* genes present in the *E. fulvus* genome, and such genes may have been lost during gene evolution.

By constructing phylogenetic trees of *EfR2R3-MYB* and *AtR2R3-MYB*, we found that S22, S25, S15, S19, S6, S3, S12, S24, and S10 contained only *AtR2R3-MYB*; E1, E2 (S21), E3, E4 (S23), E5 (S18), E6 (S20), E7, E8, E9 (S14), E10 (S1), E11 (S5), E12 (S7), E13 (S13), E14 (S16), E15 (S17), E16 (S2), E18 (S4), E19 (S11), and E20 (S9) all contained *EfR2R3-MYB* and *AtR2R3-MYB*. This finding suggests that these *R2R3-MYB* genes may have a common ancestor but underwent species-specific differentiation during the evolutionary process [51]. Gene structure is strongly linked to gene evolution and function, so we analyzed 20 motifs of the *EfMYB* gene. We found that the types and numbers of conserved motifs of *EfR2R3-MYB* genes located in the same phylogenetic tree branch are similar, which also supports our grouping. The motif composition of the *Ef1R-MYB* subgroup of genes shows diversity. In addition, we found that the number of motifs in *Ef3R-MYB* (*EfMYB26*) was the highest (16). Interestingly, the motifs of *EfMYB26* were essentially duplications of Motif 3, Motif 2, and Motif 16, and these duplicated motifs may be directly related to the function of this gene. In addition, we found that the number of introns in the *EfMYB* gene was mainly 1~2, which was similar to that reported in other plants [52,53]. However, there are also some genes that have a more prominent number of introns, such as *EfMYB12* (*EfR2R3-MYB*), *EfMYB42* (*Ef1R-MYB*), and *EfMYB26* (*Ef3R-MYB*), which contain 22, 19, and 14 introns, respectively, suggesting that *EfMYB* genes may have undergone intron loss and gain during the evolutionary process [54].

The MYB structural domain is the core region of MYB transcription factors, which can specifically bind to the promoters of target genes and thus regulate target-gene expression. We further analyzed the core motifs of the EfR2R3-MYB structural domain and found that many amino acid residues are conserved in the R2 and R3 repeat sequences. The R2 and R3 repeat motifs contained 3 and 2 conserved tryptophan residues (W), respectively (Supplementary Figure S1). The first W in the R3 repeat motif was replaced by F/I/L (phenylalanine/isoleucine/leucine), and these phenomena were also observed in *Arabidopsis*, *Morella rubra*, and *Liriodendron* [55,56,57]. In addition to the highly conserved W, Glu-11, Gly-23, Leu-36, and Arg-44 in the R2 repeat motif and Gly-25, Arg-38, and Asn-45 in the R3 repeat motif are also conserved in the EfR2R3-MYB protein. These conserved amino acid residues may together with tryptophan residues maintain the HTH structure of the MYB domain.

Gene duplication events, including whole-genome duplication (WGD), SD, and TD, are important factors driving the expansion of gene families. The occurrence of these duplication events may result in the derivation of new gene functions [58]. In this study, we found 21 segmental-duplication and 4 tandem-duplication *EfMYB* gene pairs in the whole *E. fulvus* genome, and these quantities are relatively fewer than the *MYB* duplication gene pairs in *S. spontaneum*, *Tripterygium wilfordii*, and ginger, which also explains the relatively small number of *EfMYB* genes [59,60]. It has been reported that there are usually three types of new genes derived from gene replication events, namely, nonfunctionalization, new functionalization, and subfunctionalization genes [61]. The type of *MYB* duplicated genes in *E. fulvus* needs to be further explored. Synteny analysis can be used to understand the evolutionary relationships of homologous gene pairs and to estimate the time of divergence between species [62]. In this study, *E. fulvus* and *S. bicolor MYB* homologous gene pairs were densely distributed across the chromosomes, followed by those with *O. sativa MYB* homologous gene pairs. Notably, 13 *EfMYB* genes have syntenic gene pairs with *O. sativa*, *S. bicolor*, *S. spontaneum,* and *Z. mays MYB* genes, suggesting that these genes may have undergone similar environmental selection in these species.

Several studies have shown that MYB proteins function by interacting with other family proteins. In barley, the MYB family protein (HvANT1) can further regulate the transcriptional activation of the anthocyanin synthesis-related gene *HvDFR* after binding to the bHLH family protein (HvANT2) and WD40 family protein (HvWD40-140) to form MBW complexes [63]. In protein-interaction-network analysis, we found that EfMYB proteins are tightly linked to other family proteins (ARP4, H0S15, ELF3, PRPL11, and NUP98A). Studies have shown that the NUP98A and ARP4 proteins affect flowering and leaf size in plants [64,65]. The *H0S15* gene regulates the expression of the cold-tolerance-related gene COR, thereby affecting cold tolerance in *A. thaliana* [66]. Overexpression of the *ELF3* gene makes transgenic *A. thaliana* more tolerant to salt stress [67]. PRPL11 has also been reported in studies related to plant salt-tolerance responses. [68]. These results suggest that MYB proteins may interact with these proteins and then regulate various types of responses. In addition, several studies have shown the existence of transcriptional cross-autoregulation within transcription-factor families [69]. In our study, numerous MYB proteins interacted with each other in the interaction network, suggesting that potential transcription-factor self-regulation may also exist within the *EfMYB* family.

*MYB* family genes are important in the plant adversity-stress response. Drought stress can induce expression of the *PtoMYB142* gene. Its overexpression leads to an increase in wax accumulation in poplar leaves and significantly enhances drought tolerance [70]. Drought stress also rapidly induces *AtMYB12* gene expression, and overexpressing transgenic plants accumulate more flavonoids than the wild type, which mitigates damage from drought and oxidative stress [71]. In this study, we found that *EfMYB30*, *EfMYB70*, *EfMYB81,* and *EfMYB101* were upregulated under drought stress, indicating that they may play important roles in *E. fulvus* response to drought stress. Transcriptional regulation of the plant cold-stress response involves a complex network of multiple genes. The ICE-CBF/DREB-COR cascade pathway is considered the most typical pathway regulating the cold stress response [72]. The expression of *COR* genes has been shown to play a crucial role in the plant cold-stress response. According to reports, *CBF* genes regulate only 10%-20% of genes, while various transcription factors, such as *MYB73*, *ZAT10*, *CZF2*, and *WRKY33*, can also induce the expression of *COR* genes under cold stress [73]. Studies have also indicated that the R2R2-MYB transcription factor gene *AtMYB14* can regulate the cold-stress response by modulating *CBF* gene expression [74]. *AtMYB15* can interact with *ICE1* to activate *CBF* gene expression [75]. Additionally, Li et al. found that cold stress can rapidly induce the expression of the maize gene *ZmMYB31*, and many cold-responsive genes (*AtCBF1*, *AtCBF2*, *AtCBF3*, *AtCOR1*, *AtCOR2*, *AtGSTU5*) were upregulated in transgenic lines overexpressing *ZmMYB31* [76]. Genes such as *SlMYB102*, *RmMYB108*, and *GmMYBJ1* have also been demonstrated to play positive regulatory roles in the plant cold-stress response [77,78,79]. In a previous study, we found numerous MYB binding sites in the promoter region of the *E. fulvus EfCBF/DREB* genes, indicating that the EfMYB transcription factor may play an important role in regulating *CBF* gene expression [80]. In this study, we found that *EfMYB39*, *EfMYB84*, and *EfMYB124* could be induced by low temperature very significantly using qRT-PCR analysis, suggesting that these genes may play important roles in *E. fulvus* response to cold stress. In addition, further research is needed to determine whether EfMYB transcription factors regulate *EfCBF* gene expression under cold stress.

Some studies have shown that *MYB* genes further regulate plant growth, development, and stress responses through hormone signaling pathways. For example, *PtrMYB94* responds to drought stress and ABA induction, and its overexpression upregulates some ABA and drought-responsive genes (*ABA1*, *DREB2B*) in plants and thus enhances their drought resistance [81]. *SiMYB56* enhances the drought tolerance of transgenic rice plants by regulating lignin biosynthesis and ABA signaling pathways [82]. *SiMYB75* positively regulates the responses to drought, salt, and osmotic stress through an ABA-mediated pathway [83]. *TcMYB8* and *SbMYB12* can respond to MeJA treatment [84,85], and *GlMYB4* and *GlMYB88* play important roles in the synthesis of flavonoids mediated using MeJA signaling [86]. In this study, we found that regulatory elements associated with MeJA and ABA were most common in *EfMYB*; thus, we also analyzed the expression characteristics of the nine *EfMYB* genes after ABA and MeJA treatments. *EfMYB1*, *EfMYB30*, *EfMYB39*, *EfMYB84*, and *EfMYB130* responded positively to ABA treatment. Among them, EfMYB30 also had a positive response to drought stress, indicating that the gene may be involved in the regulation of drought stress in *E. fulvus* through ABA signaling. In addition, we found that these nine *EfMYB* genes respond to MeJA treatment to varying degrees. In this study, the gene structure and evolutionary characteristics of *EfMYB* family genes were comprehensively studied, and *EfMYB* genes with potential functions in drought and cold resistance were identified. We will study the function and regulatory pathway of these candidate genes in future research.

## 5. Conclusions

In this study, a total of 133 *EfMYB* genes were identified in the whole *E. fulvus* genome, and phylogenetic, gene structure, evolutionary, protein-interaction, and expression-characterization analyses were performed to illustrate the structural features, possible evolutionary mechanisms, and potential functions of the gene family members. The results of this study provide a good basis for further study of the biological function and regulatory mechanism of the *EfMYB* gene and the genetic improvement of transgenic sugarcane.

## Data Availability

The data are contained within the article or Supplementary Files.

## Supplementary Materials

The following supporting information can be downloaded at https://www.mdpi.com/article/10.3390/genes14122128/s1, Supplementary Figure S1: Conserved motifs in the R2R3-MYB structural domains; Table S1: EfMYB protein sequence; Table S2: Physicochemical information and subcellular localization of 133 *EfMYB* genes; Table S3: *EfMYB* duplicated gene pairs; Table S4: Twenty conserved motifs obtained by MEME software (Version 5.5.4); Table S5: One-to-one orthologous relationships between *E. fulvus* and the other five plant species; Table S6: Cis-acting elements of the promoter region of *EfMYB* family genes; Table S7: Detailed information of the interaction network of EfMYB family proteins; Table S8: Primers, system and procedure for qRT-PCR in this study.
