# Supplementary figures and images for: Genome-Wide Identification and Expression Pattern of MYB Family Transcription Factors in Erianthus fulvus
Source: Genes (Basel). 2023 Nov 25;14(12):2128. doi: 10.3390/genes14122128 (PMC10743048; doi:10.3390/genes14122128)

a

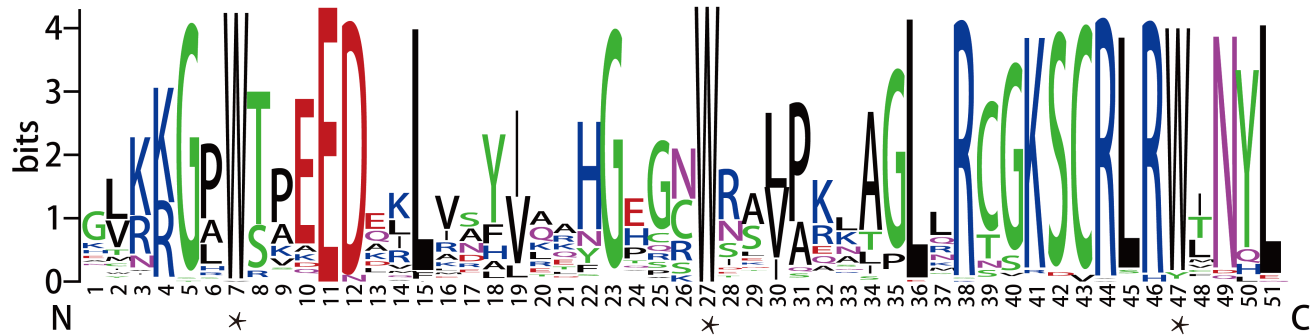

b

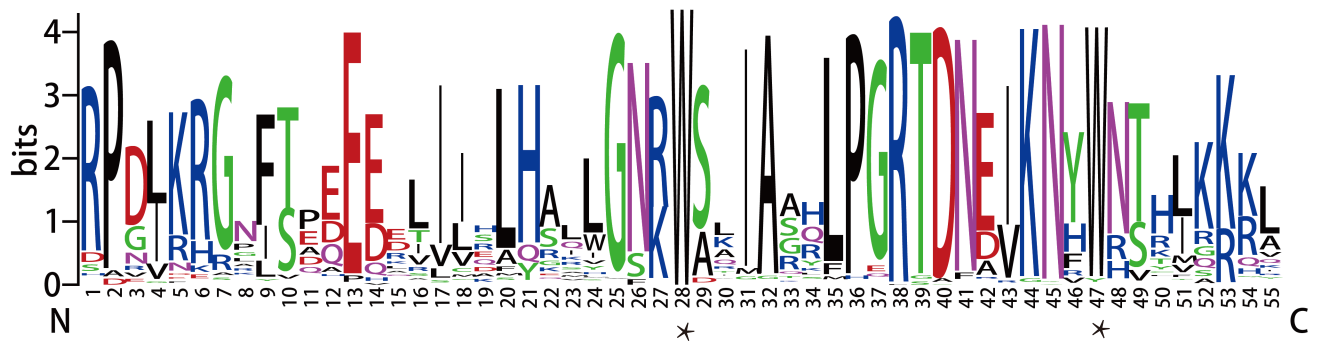

**Figure S1.** R2(a) and R3(b) repeats of the proteins of the R2R3-MYB family in *Erianthus fulvus*

Supplement: Supplementary file 1 [file genes-14-02128-s001.zip › Figure S1.pdf]
